# Relation of Dietary Practices and Academic Achievement Among School-Going Children in Kattankulathur Block, Chengalpattu District, Tamil Nadu

**DOI:** 10.7759/cureus.72882

**Published:** 2024-11-02

**Authors:** Ashmitha Rajan, Palani Vel. M, Roshni Mary Peter, Logaraj M, Anantharaman V V

**Affiliations:** 1 Community Medicine, SRM Medical College Hospital and Research Centre, SRM Institute of Science and Technology, Chengalpattu, IND

**Keywords:** academic achievement, dietary practices, nutritional education, school children, school performance

## Abstract

Background

Dietary practices play a crucial role in the overall development of school-going children, potentially influencing their academic performance. Nutrition is a fundamental component of children's overall health and development, influencing both physical growth and cognitive functions. School-going children are particularly vulnerable to the effects of their diet, as it impacts their ability to concentrate, learn, and perform academically.

Methodology

A cross-sectional study was conducted with 262 schoolchildren aged 11-16 years from various schools in Kattankulathur Block, Chengalpattu District. Dietary practices were assessed using a pretested questionnaire, while academic performance was evaluated based on school records. Data analysis included a t-test, as well as correlation and regression analyses, to examine the relationships between the dependent and independent variables. The data were analyzed using IBM SPSS (IBM SPSS Statistics for Windows, IBM Corp., Version 26, Armonk, NY).

Results

The study found a significant correlation between balanced dietary practices, including regular intake of fruits and vegetables, and higher academic achievement. Studies show that 88.2% of children have three regular meals daily, 88.9% have breakfast consistently, and 24.8% of children have excellent school performance. Children with poor dietary habits exhibited lower academic performance compared to those with healthier diets.

Conclusion

Nutritional practices significantly impact academic performance among school children. Improving dietary habits can enhance cognitive functions and academic outcomes, emphasizing the need for better nutritional education and interventions in schools.

## Introduction

Globalization, urbanization, and economic development have led to considerable transformations that have impacted various aspects of everyday life, including lifestyle changes. Notably, eating habits have evolved over the last few decades, characterized by an increase in the consumption of fats, particularly saturated fats, alongside a decrease in the intake of grains, fruits, legumes, and vegetables, which is reflective of a fast-paced lifestyle [1,2]. Breakfast is crucial for memory retention, recognition, and mental strength needed for daily activities. Academic success can be influenced by several factors, such as socioeconomic status, gender, ethnicity, and the quality of education and school experience. Skipping meals, especially breakfast, often results in poorer dietary choices later in the day, which can adversely affect learning and performance in academics [3,4].

Dietary choices may play a role in the occurrence and severity of overweight and obesity among children and adolescents. The trend of skipping breakfast has become more prevalent in these age groups and is associated with increased measures of body fat [5,6]. A healthy diet is essential for mental well-being, as it significantly affects school performance. Thus, previous research indicates a strong correlation between high consumption of sugar, fats, and fast foods, leading to poor academic outcomes and various health issues, including metabolic diseases in children [6,7]. Breakfast is particularly significant for students, as it supplies essential micronutrients - such as vitamins, carbohydrates, and minerals - that are vital for brain health and cognitive functions [8]. Children and adolescents with unhealthy eating patterns face a heightened risk of developing chronic conditions like diabetes, dyslipidemia, and hypertension in adulthood (World Health Organization (WHO) 2000) [8].

A common reason for poor nutrition behaviors is a lack of nutritional knowledge (9). The WHO has previously recommended that health professionals should actively encourage healthy eating practices and provide nutritional care. General practitioners (GPs) offer comprehensive medical care to individuals and families within their communities. Given their regular interaction with patients, GPs are expected to deliver health-related information [9,10].

This study aimed to assess the impact of dietary practices on the academic achievement of school-going children and to identify the association between nutritional habits and academic performance in this population in the Kattankulathur Block, Chengalpattu District, Tamil Nadu. The objectives of this study are to assess the dietary habits of school-going children in Kattankulathur Block, including the type, frequency, and nutritional quality of their food, to evaluate the academic performance of school-going children based on their grades, participation in school activities, and to examine the relationship between specific dietary practices (e.g., breakfast consumption, intake of fruits and vegetables, junk food consumption) and academic performance.

## Materials and methods

A cross-sectional study was carried out in the Chengalpattu region, targeting schoolchildren aged 11 to 16 years, with data collection occurring from July 2023 to March 2024. To ensure fairness in participant selection, 262 schoolchildren were picked using a simple random sampling method. About 18 schools, comprising seven primary and 11 secondary schools, are located in the Kattankulathur block. Among these 11 schools, six secondary schools were chosen at random. Then, from the six previously chosen schools, three were chosen at random using a probability proportional to size sampling technique. The final sample size of 262 children aged 11 to 16 was derived from a total of 864 students throughout all three of these schools. The research received ethical approval from the ethics committee of the host institution under reference number 2073/IEC/2020. The study strictly adhered to ethical guidelines, particularly in relation to participant selection and consent processes. Participants were chosen based on specific criteria that mandated they reside within the Kattankulathur block and be fluent in either Tamil or English. Additionally, all participants were required to confirm their willingness to provide informed consent and to engage in the assessment sessions. Exclusion criteria were also established, disqualifying students who opted out of participation or were unable to supply the necessary data. To maintain ethical standards, informed consent was obtained from the parents or legal guardians of all participants before any data collection began, ensuring that ethical guidelines were respected and that the participants' rights and welfare were safeguarded throughout the study.

Data collection involved structured face-to-face interviews, which promoted direct interaction and ensured clarity in the responses of the participants. A pre-tested questionnaire was utilized during these interviews, specifically designed to reduce ambiguity in both the questions and the responses. The pre-testing of the questionnaire helped enhance its reliability and validity, ensuring that the collected data was accurate and could be used to draw meaningful conclusions. The structured nature of the interviews and the validated questionnaire contributed to the robustness of the data collection process, guaranteeing that the gathered information was comprehensive and reliable for further analysis.

The questionnaire was divided into two key sections:

The first section gathered socio-demographic data, health status, and family background information of the participants, providing a contextual understanding of their living conditions and family dynamics.

The second section focused on nutritional status and dietary plan, covering aspects such as breakfast consumption, meal frequency, and the prevalence of fast food consumption among the students. This section also sought to capture any significant patterns or trends in dietary habits.

Following the data collection, the responses were carefully reviewed, coded, and entered into IBM SPSS (IBM SPSS Statistics for Windows, IBM Corp., Version 26, Armonk, NY) to ensure accuracy and completeness in the entry process. A p-value less than or equal to 0.05 is considered statistically significant. For descriptive analysis, the distribution of each socio-demographic characteristics, dietary, and academic performance variable was calculated and presented as both frequencies and percentages. To explore potential associations between students' dietary habits and their academic performance, relevant statistical tests were employed. The chi-square test of independence was primarily used to assess the relationships between categorical variables, providing insights into whether eating behaviors had a significant impact on students' academic outcomes. Correlation and regression analyses were conducted to assess the relationship between dietary habits and academic performance.

Operational definitions

Dietary practices: The consistent eating habits or patterns, including the frequency of meals, types of food consumed (e.g., breakfast, lunch, snacks), and nutritional content (e.g., proteins, vitamins, fats, and carbohydrates) [10].

School performance: The measurable outcomes of a student's educational achievements, often assessed through grades, standardized test scores, and other evaluations of academic knowledge and skills [10].

## Results

Table 1 shows that the majority of the 262 youngsters polled (66%) are between the ages of 13 and 16, with a substantial representation of males (58%), according to the socio-demographic statistics. First-born children make up 49.6% of the birth order. When it comes to family income, half of them make between Rs.10,000 and Rs. 20,000, while a sizable fraction (31.3%) make less than Rs. 10,000. Fathers have a lower educational background than women: 46.2% of fathers have completed secondary education, compared to 53.4% of mothers. Compared to 64.9% of mothers, a huge majority of fathers (93.9%) are working, and virtually all children (94.3%) have their moms take primary care of them.

**Table 1:** Socio-demographic characteristics of school children

Child socio-demographic data		Frequency (n = 262)	Percentage (%)
Age in years	11-13 years	89	34
	14-16 years	173	66
Gender	Male	152	58
	Female	110	42
Child order	First	130	49.6
	Middle	71	27.1
	Last	61	23.2
Family income	<10,000	82	31.3
	10,000-20,000	131	50
	>20,000	49	18.7
Fathers education	Primary	114	43.5
	Secondary	121	46.2
	Degree	27	10.3
Mothers education	Primary	92	35.1
	Secondary	140	53.4
	Degree	30	11.5
Fathers work	Working	246	93.9
	Not working	16	6.1
Mothers work	Working	170	64.9
	Not working	92	35.1
Child caregiver	Father	9	3.2
	Mother	268	94.3
	Others	7	2.5

Table 2 shows that 262 children's eating patterns and academic achievement typically show upward increases in both areas of nutrition and involvement; 88.2% of respondents say they eat three meals a day on average, and 88.9% say they eat breakfast every day. While a sizable 82.8% of children eat fruits and vegetables, the majority of children, 72.9%, also snack in between meals. It is interesting to note that 66.4% of respondents eat fast food one to three times per week, and 51.1% prefer it to homemade meals; 59.9% of students evaluated their academic performance during the previous three months as ordinary, while 24.8% performed exceptionally well. There was a high rate of student attendance 82.1% attended classes well (above 75%).

**Table 2:** Dietary habits and school performance of children

Dietary habits		Frequency(n = 262)	Percentage (%)
Have three meals regularly	Yes	231	88.2
	No	31	11.8
Child has breakfast only	Yes	233	88.9
	No	29	11.1
Snacks between meals	Yes	191	72.9
	No	71	27.1
Child eats fruits and vegetables	Yes	217	82.8
	No	45	17.2
Child prefer fast food to homemade food	Yes	134	51.1
	No	128	48.9
Frequency of fast food/week	Never	85	32.4
	One to three days/week	174	66.4
	Daily	3	1.1
School performance in last three months	Poor (<50%)	40	15.3
	Fair (50-75%)	157	59.9
	Good (>75%)	65	24.8
School attendance in last three months	Poor (<50%)	3	1.1
	Fair (50-75%)	44	16.8
	Good (>75%)	215	82.1

Table 3 shows that the association between children's eating habits and their academic achievement is analyzed. For children who regularly eat three meals a day, the chi-square value for the chance of academic performance is 6.51 (p = 0.03). Additionally, breakfast eaters do better, as evidenced by a chi-square value of 7.10 (p = 0.02). Consuming fruits and vegetables also has a positive association (chi-square = 10.72, p = 0.005) with academic success. Fast food preferences, on the other hand, have a chi-square value of 10.28 (p = 0.006), which indicates a negative association with performance. The consumption frequency of fast food indicates that individuals who never consume it outperform others (chi-square = 10.82, p = 0.02).

**Table 3:** Association between school children’s eating habits and their school performance *p-value < 0.05 is statistically significant.

School performance						
Eating habits		Poor	Fair	Good	Chi-square value	p-value
		Frequency (%)	Frequency (%)	Frequency (%)		
Have three meals regularly	Yes	31 (13.5%)	144 (62.3%)	56 (24.2%)	6.51	0.03*
	No	9 (29%)	13 (41.9%)	9 (29%)		
Child has breakfast only	Yes	32 (13.7%)	146 (62.6%)	55 (23.6%)	7.10	0.02*
	No	8 (27.5%)	11 (37.9%)	10 (34.4%)		
Child eats fruits and vegetables	Yes	26 (11.9%)	134 (61.9%)	57 (26.2%)	10.72	0.005*
	No	14 (31.1%)	23 (51.1%)	8 (17.7%)		
Child prefer fast food to homemade food	Yes	16 (11.9%)	93 (69.4%)	25 (18.6%)	10.28	0.006*
	No	24 (18.7%)	64 (50%)	40 (31.2%)		
Frequency of fast food/week	Never	7 (8.2%)	48 (56.4%)	30 (35.2%)	10.82	0.02*
	One to three times/week	32 (18.3%)	107 (61.4%)	35 (20.1%)		
	Daily	1 (33.3%)	2 (66.6%)	0 (0%)		

Table 4 shows that analysis with school performance as the constant variable reveals that consuming fruits and vegetables explains 2.76% (r = 0.166, p = 0.007) of the variance in better academic outcomes, and maintaining good attendance explains 2.89% (r = 0.170, p = 0.006). Frequent fast food consumption negatively impacts academic outcomes, explaining 4.08% (r = -0.202, p = 0.001) of the variance. In contrast, variables like having three meals regularly, daily breakfast, and snacking between meals show weak or non-significant correlations, indicating that these habits do not significantly influence academic success.

**Table 4:** Correlation matrix of dietary habits, school performance, and attendance *p-value < 0.05 is statistically significant.

Variables	Three meals regularly	Has breakfast daily	Snacks between meals	Eats fruits and vegetables	Prefers fast food	Fast food frequency	School performance	Attendance (past three months)
Three meals regularly	1	0.549*	0.069	0.146*	-0.051	0.007	0.056	0.002
Has breakfast daily	-	1	0.004	0.065	-0.077	-0.027	0.015	-0.102
Snacks between meals	-	-	1	0.041	0.005	0.102	-0.031	0.132*
Eats fruits and vegetables	-	-	-	1	-0.060	-0.064	0.166*	0.106
Prefers fast food	-	-	-	-	1	0.469*	-0.046	-0.098
Fast food frequency	-	-	-	-	-	1	-0.202*	0.044
School performance	-	-	-	-	-	-	1	0.170*
Attendance (past three months)	-	-	-	-	-	-	-	1

Table 5 shows significantly that eating more fruits and vegetables is positively correlated with improved performance (standardized coefficient: 0.132; p = 0.03). Furthermore, there is a negative correlation (coefficient of -0.243; p = 0.001) between the frequency of fast food consumption and performance. There is also a positive link (β = 0.182, p = 0.003) between attendance over the previous three months. On the other hand, p-values of 0.98 and 0.53, respectively, show that eating regularly and eating breakfast every day do not significantly affect academic outcomes. These findings emphasize how crucial a balanced diet and regular attendance are to academic success.

**Table 5:** Multiple linear regression analysis of factors affecting school performance in the last exam *p-value < 0.05 is statistically significant.

Model	Standardized coefficients beta (β)	t	p-value	95% confidence interval	
				Lower bound	Upper bound
(Constant)					
Have three meals regularly	0.045	0.61	0.53	-0.18	0.36
Child has breakfast daily	0.001	0.02	0.98	-0.28	0.28
Snacks between meals	-0.039	-0.64	0.52	-0.22	0.11
Child eats fruits and vegetables	0.132	2.18	0.03*	0.02	0.41
Child prefers fast food to home food	0.096	1.41	0.16	-0.04	0.28
Frequency of fast food/week	-0.243	-3.56	0.001*	-0.48	-0.14
Attendance in the past three months	0.182	2.96	0.003*	0.09	0.45

## Discussion

The socio-demographic analysis of the 262 children in the study reveals that 66% are aged between 13 and 15 years, with a slight male predominance (58%). Half of the participants are first-born, and nearly 50% of their families have an annual income ranging from Rs. 10,000 to Rs. 20,000. Comparatively, McLoyd et al. [11] emphasize the significant impact of socioeconomic status, particularly family income, on a child's educational opportunities. The majority of children studied fall within the 13-15 age range, aligning with Burchinal et al. [12] findings that the early stages of adolescence are crucial for educational development. In this research, only a small fraction of the children have three meals a day, while the majority consistently have breakfast. Rampersaud et al. [13] found that most children who regularly eat breakfast tend to show improved dietary quality and cognitive performance. Mullan and Singh [14] reported that about 85% of children consume three meals daily, indicating a strong association between regular meal intake and better academic results and health outcomes. In our study, 72.9% of the children reported snacking between meals. A study by Nicklas et al. [15] noted that 83% of children snack between meals, with the types of snacks consumed (which are often high in fat, sugar, and salt) potentially compromising nutritional quality. However, healthy snacks like fruits and vegetables can foster beneficial dietary habits.

According to this report, 82.8% of children consume fruits and vegetables. However, only 40% of children aged one to 18 meet the daily recommended consumption of fruits and vegetables, as per a study from the Centers for Disease Control and Prevention (CDC) published in 2017 [16]. Conversely, a different study by Evans et al. [17] established that 75-80% of children regularly eat fruits and vegetables. In our research, 51.1% of children prefer fast food over home-cooked meals, with 66.4% consuming fast food one to three times a week. Bowman et al. [18] noted that approximately 30% of children eat fast food daily, with 47% expressing a preference for it over homemade meals. This study strongly linked fast food consumption to increased calorie intake, lower nutritional quality, and a higher risk of obesity. Our findings indicate that 24.8% of children achieved exceptional academic performance (greater than 75%), while 59.9% performed at an average level (between 50 and 75%). Studies show that children who maintain a balanced diet and regular meal patterns are more likely to excel academically; indeed, nearly 75% of children in schools prioritizing nutrition performed at average or above levels [19]. Additionally, 82.1% of children exhibited outstanding attendance rates (greater than 75%). Research by Lykens et al. [20] demonstrated that children with healthier eating habits tend to have higher school attendance rates, with 75-80% of those practicing good nutrition achieving similar attendance records.

Consistently eating three meals a day was found to have a substantial positive correlation with academic success, suggesting that such regularity enhances performance. Hoyland et al. [21] study further noted that meal skipping correlates with poorer academic performance, especially among younger children. Children who eat breakfast consistently are more likely to perform well or on average. Adolphus et al. [22] indicate that regular breakfast consumption is associated with improved academic performance due to better memory, attention, and problem-solving skills. Our research found that children eating fruits and vegetables regularly achieve better academic results. Similar evidence is provided by Storey et al. [23], suggesting that diets rich in fruits and vegetables are tied to enhanced cognitive function and educational success, as these foods are packed with essential nutrients that support overall health and, consequently, cognitive performance. Children who favor fast food over home-cooked meals tend to perform worse academically, a finding supported by Poti et al. [24], who noted frequent fast-food consumption is linked to lower test scores and attention issues in the classroom. Furthermore, this study found that children who abstain from fast food perform better academically. Li et al. [25] confirmed that children who frequently consume fast food encounter more academic challenges. The intake of fast food has been associated with suboptimal nutritional status, impacting mental capability and academic performance.

The results from this study indicated no significant correlation between the consistent consumption of three meals and academic performance (r = 0.056, p = 0.368). Similarly, Burrows et al. [26] found that children who regularly have meals are more likely to excel academically due to constant nutritional intake. Irregular eating patterns may negatively influence attention span and cognitive functioning, as suggested by Leech et al. [27]. In this study, no significant relationship was found between regular breakfast consumption and academic performance (r = 0.015, p = 0.810). Similarly, a study by Wang et al. [28] shows no significant association between regular breakfast consumption and academic performance in their research, and Mugridge et al. [29] also found no definitive link between breakfast intake and academic achievement. Our study showed no significant correlation between snacking and academic performance (r = -0.031, p = 0.623). This aligns with findings by Tahmassebi et al. [30], which linked frequent unhealthy snacking to poorer academic outcomes, especially among those consuming sugary snacks. Conversely, St-Onge et al. [31] found that healthy snacks, like fruits and nuts, positively influence cognitive performance by providing sustained energy between meals.

## Conclusions

The study's findings indicate that while most students demonstrate strong academic performance and comprehension skills, nearly half report facing challenges in their studies, and a small subset shows low performance. Although absenteeism related to dietary habits is minimal, it highlights the need for enhanced nutritional support to address specific difficulties. Overall, the high school attendance rates suggest positive student engagement; however, targeted interventions are necessary to improve educational outcomes and well-being further. This underscores the crucial role of nutrition, as well-nourished students consistently perform better academically. Initiatives like India’s Midday Meal Scheme illustrate the impact of improved nutrition on educational success, making it essential to continue focusing on dietary support for students to ensure their academic and personal development. The limitations of this study include, first, the reliance on self-reported data from children or parents regarding dietary habits and academic performance, which may introduce reporting biases or inaccuracies, particularly in recalling specific meals or practices. Additionally, the cross-sectional design of the study limits the ability to establish a causal relationship between dietary habits and academic achievement, as it only provides a snapshot in time. Confounding factors such as socio-economic status, parental education, and access to educational resources could also influence both dietary practices and academic performance, making it difficult to isolate the direct effects of diet. The sample size and geographic focus of the study may further limit the generalizability of the findings to other regions or populations. Moreover, the study might not assess the nutritional quality of the meals consumed, focusing only on the frequency or specific practices, which could be more closely linked to cognitive function. School-related factors, such as differences in infrastructure and teaching quality, were not controlled for, potentially affecting academic outcomes independently of diet. Lastly, the short-term measurement of academic performance may not capture the long-term effects of dietary practices on learning and achievement, limiting the scope of the findings.
